# A CALL TO ACTION FOR FALL INJURY PREVENTION: QUALITY IMPROVEMENT IN THE DEPARTMENT OF VETERANS HEALTH ADMINISTRATION

**DOI:** 10.1093/geroni/igad104.2268

**Published:** 2023-12-21

**Authors:** Blake Barrett, Margeaux Chavez, Sarah Bradley, Nora Arriola, Vianna Broderick, Anita Ramrattan, Tatjana Bulat, Linda Cowan

**Affiliations:** VISN 8. Patient Safety Center of Inquiry, James A. Haley Veterans’ Hospital and Clinics, Department of Veterans Health Administration, Tampa, Florida, United States; VISN 8. Patient Safety Center of Inquiry, James A. Haley Veterans’ Hospital and Clinics, Department of Veterans Health Administration, Tampa, Florida, United StatesStates; VISN 8. Patient Safety Center of Inquiry, James A. Haley Veterans’ Hospital and Clinics, Department of Veterans Health Administration, Tampa, Florida, United StatesStates; VISN 8. Patient Safety Center of Inquiry, James A. Haley Veterans’ Hospital and Clinics, Department of Veterans Health Administration, Tampa, Florida, United StatesStates; VISN 8. Patient Safety Center of Inquiry, James A. Haley Veterans’ Hospital and Clinics, Department of Veterans Health Administration, Tampa, Florida, United StatesStates; VISN 8. Patient Safety Center of Inquiry, James A. Haley Veterans’ Hospital and Clinics, Department of Veterans Health Administration, Tampa, Florida, United StatesStates; VISN 8. Patient Safety Center of Inquiry, James A. Haley Veterans’ Hospital and Clinics, Department of Veterans Health Administration, Tampa, Florida, United StatesStates; VISN 8. Patient Safety Center of Inquiry, James A. Haley Veterans’ Hospital and Clinics, Department of Veterans Health Administration, Tampa, Florida, United States

## Abstract

The rates of inpatient falls in the Department of Veterans Health Administration (VHA) have been declining steadily over the last 10 years, but the rates of major fall-related injuries have remained stagnant. This poster describes quality improvement (QI) efforts in the VHA to evaluate and improve implementation of fall injury prevention practices. It also describes the importance of focusing on fall injury prevention, in addition to fall prevention. QI efforts were led by an interdisciplinary team using mixed methods. Objectives included: 1) determine best practices in fall injury prevention in VHA inpatient units, 2) determine the utility of current methods for evaluating implementation of fall injury prevention practices, and 3) improve fall injury prevention practice implementation by co-designing an evaluation tool with VHA staff who are its intended users. Findings suggest the need to raise awareness of and use of fall injury prevention practices, that is distinct from fall prevention practices. Staff do not typically separate fall injury prevention practices from general fall prevention, making it challenging to implement tailored prevention practices (e.g., hip protectors or helmets directed at fall injury prevention vs. fall prevention). Furthermore, staff reported difficulty implementing and evaluating fall injury prevention practices. Staff preferred tools using a formal evaluation process with interdisciplinary teams to outline standards and document barriers and facilitators to reducing fall injuries. Our findings support need for a validated tool to evaluate and improve implementation of fall injury prevention practices (above and beyond fall prevention) to improve care and reduce Veteran fall-related injuries.

